# Safe and unsafe urinary catheter use in long-term care homes: a human factors-informed scoping review protocol

**DOI:** 10.3389/fmed.2026.1939120

**Published:** 2026-09-30

**Authors:** Sai Prashanti Mohan, Sneha Negi, Om Patel, Maya Pot, Khadija Aliyeva, Adeena Qudrat, Rayyan Qudrat, Lucas Scarvelis, Heather MacDonald, Samantha Boulerice, Benoît Robert, Chantal Trudel, Adrian D. C. Chan, Atul Jaiswal

**Affiliations:** 1 Department of Systems and Computer Engineering, Carleton University, Ottawa, ON, Canada; 2 Department of Health Sciences, Carleton University, Ottawa, ON, Canada; 3 Department of Health Sciences, McMaster University, Hamilton, ON, Canada; 4 Department of Political Science, Dalhousie University, Halifax, NS, Canada; 5 Department of Health Sciences, University of Ottawa, Ottawa, ON, Canada; 6 Perley Health, Ottawa, ON, Canada; 7 Department of Psychology, Queens University, Kingston, ON, Canada; 8 Research Support Services, Carleton University, Ottawa, ON, Canada; 9 School of Industrial Design, Carleton University, Ottawa, ON, Canada

**Keywords:** catheter related infections, ergonomics, long term care, practice guideline (MeSH), scoping review, urinary catheter, urinary tract infections

## Abstract

**Introduction:**

Transurethral catheters are a type of urinary catheters that are commonly used in long-term care (LTC) settings to provide wound healing assistance, manage urinary retention and acute urinary obstruction. However, inappropriate and prolonged catheter use increases the risk of complications such as catheter-associated urinary tract infections (CAUTIs), blockages, leakage, trauma and discomfort, which negatively affects resident outcomes. In the LTC context, CAUTIs lead to increased morbidity, prolonged stay in hospitals and overall higher healthcare costs. Although clinical practice guidelines exist to support transurethral catheter management, evidence specific to LTC settings remains limited and has largely focused on clinical outcomes, with less attention given to the human factors and work system conditions that influence catheter-related care. Guided by the Systems Engineering Initiative for Patient Safety (SEIPS) framework, this scoping review aims to map and synthesize the existing evidence on transurethral catheter use in LTC homes, identify evidence-informed management practices, and examine the work system factors that influence safe and appropriate catheter use.

**Methods:**

The Joanna Briggs Institute (JBI) methodology will be followed for the scoping review in adherence to the PRISMA-ScR guidelines. A preliminary search was conducted in PROSPERO, MEDLINE and Cochrane Databases of Systematic review to refine and review terms, followed by comprehensive and final searches across multiple databases, including PubMed, CINAHL, Embase, MEDLINE, Web of Science and SCOPUS. Study selection will be completed using the Covidence systematic review software and eligible studies will focus on urinary catheter use and management in LTC globally with no date restriction. Studies will be included if full texts are available in English or French. The extracted and synthesized data will then be characterized thematically to identify themes and knowledge gaps.

**Discussion:**

This scoping review will provide an extensive literature synthesis while identifying gaps in practices to enhance catheter management in LTC settings. Guided by the SEIPS framework, this review will examine how interactions among people, tasks, technology, the physical environment, and organizational factors influence transurethral catheter management.

**Ethics and dissemination:**

This review acts as a knowledge synthesis tool based on existing literature and thus does not require any ethics approval. Summaries and findings can be used to guide future policies, guidelines, and healthcare reforms.

## Introduction

Urinary catheters are commonly used medical devices in the Long-Term Care (LTC) setting to manage urinary retention, acute urinary obstruction, and for wound management (1–3). In the United States, there are 15,700 LTC homes housing nearly 1.5 million residents (4). Among these residents, the prevalence of urinary catheterization ranges from 5%-22% (4). In Canada, there are 2,076 LTC homes, with 626 located in Ontario, housing nearly 78,000 residents (5, 6). Within Canadian LTC homes, urinary catheter use ranges from 5%-10% (7).

There are three main types of urinary catheters: external, urethral, and suprapubic (1, 8). External catheters are attached to the external genitalia in the form of a urine pouch, or a penile shaft catheter, and are the least invasive (1, 9). Urethral catheters are inserted directly into the bladder making them more invasive, and these can either be intermittent or indwelling (1, 3, 8). Intermittent catheters are frequently recommended to individuals who have difficulty fully emptying their bladder (10). Once the bladder is fully emptied or decompressed, the catheter is removed, and if necessary, reinserted at regular intervals based on individual needs (1). Indwelling catheters, in contrast, are used in the case of acute urinary retention, urinary obstruction, or to manage wound healing in chronically ill patients (1, 3). Suprapubic catheters are the most invasive as they require surgical insertion through the abdominal cavity directly into the bladder (1). These are typically used to facilitate bladder emptying when other alternatives including intermittent catheterization, are not feasible or advisable (11).

A recent systematic review reported that the prevalence of indwelling catheters in the LTC setting among different countries (including Germany, United Kingdom, Sweden, and United States of America) ranges from 2.2% to 36.4%, making indwelling catheters the most common type of catheter used in the LTC setting (2, 3, 12). However, prolonged catheter use (more than 1 month) increases risk of complications such as catheter-associated urinary tract infections (CAUTIs), urine leakage, urethral trauma, catheter blockage, or encrustation (3, 13). The improper use of indwelling catheters for managing urinary incontinence without adhering to proper guidelines can result in antibiotic-resistant infections (caused due to biofilm formation), contributing to higher morbidity and mortality rates, extended hospital stays, and increased healthcare costs (1, 2, 33). Due to these risks, indwelling catheters should be used only under certain conditions including (i) bladder emptying in terminally ill patients, (ii) monitoring urine output in critically ill patients, (iii) managing acute urinary obstruction or retention (iv) aiding wound healing in individuals with urinary incontinence (3). Guidelines from the United States of America, United Kingdom and China emphasize the importance of limiting indwelling catheter use, recommending removal when no longer necessary and favoring less invasive alternatives whenever possible (3).

Despite these recommendations, managing the use of indwelling catheters in LTC homes is complicated due to the residents’ coexisting health conditions and limited staff training (14). Since some clinical indicators may persist over time, healthcare providers may prolong the use of indwelling catheters beyond the recommended duration (2, 3). In the Canadian LTC context, catheters need to be changed every 30 days regardless of clinical symptoms (including infection, urine leakage, catheter blockage or encrustation) or before the 30-day period if clinical symptoms occur (30). However, infection control guidelines report a contrasting approach, recommending that long-term catheters should be changed only when clinically indicated, and not on a routine schedule, in order to minimize the risk of CAUTIs (30, 31). Such contradictory recommendations highlight the need for standardized protocols for catheter prescription, insertion, maintenance, change and removal to ensure that catheters are only used when necessary and are replaced in a timely manner (15, 16, 30, 31). Canadian LTC homes operate under different funding and ownership models, including publicly funded and privately owned homes (17, 18). Despite the widespread use of transurethral catheters in LTC homes, no standardized protocols exist in the Canadian LTC context to manage prolonged catheterization, to the best of our knowledge (1, 16).

While numerous guidelines describe recommended transurethral catheter practices, comparatively little attention has been paid to the human factors and work system conditions that influence whether these practices can be successfully implemented within LTC homes. Catheter-related outcomes are shaped not only by clinical decision-making but also by complex interactions among healthcare providers, residents, organizational policies, technologies, care processes, and the physical environment (32). Consequently, understanding transurethral catheter management from a systems perspective may provide insights beyond traditional infection prevention approaches.

To address this gap, this scoping review will be informed by the Systems Engineering Initiative for Patient Safety (SEIPS) model, a human factors framework that examines how interactions among people, tasks, technologies, the physical environment, and organizational factors influence healthcare processes and outcomes (32). Within the context of transurethral catheter management, the framework enables consideration of multiple interacting components, including residents and healthcare providers (people), catheter-related activities such as insertion, maintenance, removal, change, and monitoring (tasks), transurethral catheters and associated equipment (technology), the LTC care environment (physical environment), and other considerations such as staffing, policies, communication, and workflow (organizational factors). Examining catheter use through this systems-based perspective may help identify barriers and facilitators to safe catheter management that may not be evident when focusing solely on clinical practice guidelines.

Although substantial evidence exists regarding urinary catheter management in acute care and other healthcare settings, the evidence specific to LTC remains limited. Furthermore, previous reviews have primarily focused on clinical outcomes, infection prevention, or guideline adherence, with limited consideration of the human factors and work system characteristics that influence catheter-related care. By synthesizing the available evidence through a human factors lens, this review will map not only evidence-based catheter management practices but also the interactions among people, technology, tasks, the care environment, and organizational systems that influence their implementation. This systems-oriented approach has the potential to identify context-specific opportunities for improving catheter design, clinical workflows, staff education, organizational policies, and ultimately the safety and quality of care for LTC residents.

In this review, safe and unsafe transurethral catheter use will be examined based on how these practices are described in the existing literature. The review will not independently classify practices as safe or unsafe but will map and synthesize how these concepts are reported in the available evidence.

A preliminary search of PROSPERO, MEDLINE and Cochrane Database of Systematic Review found no comprehensive reviews focusing on evidence-based strategies to manage catheter use in LTC homes.

### Review objective

Through this scoping review, we aim to map the existing evidence on the prevalence, outcomes, and key factors associated with urinary catheter use in LTC homes. This will help us understand what is happening in the context of urinary catheter use in LTC homes. Using SEIPS model as an analytical framework, we will then examine how interactions among people, tasks, technology, the physical environment, and organizational factors influence the safe and unsafe use and management of urinary catheters. This will enable us to identify the potential reasons of why and how various aspects of catheter management are influenced in the context of LTC homes, highlight gaps in the existing evidence, and identify opportunities for future research. Finally, we will synthesize evidence on recommended practices across the various aspects of catheter management, including prescription, design and selection, insertion, maintenance, changing, and removal, to identify approaches that may support safer and more appropriate catheter use in LTC homes.

### Review question (s)

1. What does the existing evidence report about the prevalence, outcomes, and key factors influencing urinary catheter use in the LTC homes?
2. What human factors and work system characteristics (such as people, tasks, technology, physical environment, and organizational factors) influence the safe and unsafe use and management of urinary catheters in LTC homes?
3. What practices have been reported to support safe and appropriate prescription, design, selection, insertion, maintenance, removal and changing of indwelling urinary catheters in the LTC homes?

### Inclusion criteria

#### Participants

This review will include studies that focus on the use of urinary catheters in LTC homes (also referred as nursing homes). Studies focusing on LTC home residents, who are 18 years or older, will be included (Table 1). A transurethral catheter is a hollow tube inserted into the bladder to drain and collect urine (19). The Canadian Institute of Health Information defines LTC homes as facilities that provide a wide range of health and personal care services for individuals with medical or physical needs who require access to 24-hour nursing care, personal care, and other therapeutic and support services (17). For the purpose of this review, we will focus specifically on LTC homes that provide nursing, personal, and supportive care to residents on a long-term basis. Settings that primarily provide short-term rehabilitation or recovery services, including sub-acute rehabilitation centres, will be excluded. Studies related to the design, prescription, selection, insertion, maintenance, change and, removal of urinary catheters will be included when they specifically focus on the residents of LTC homes. Studies focusing solely on CAUTIs or HAIs or UTIs, in settings other than LTC homes, will not be included.

**Table 1:** Inclusion and exclusion criteria.

Included when	Exclusion when
The focus is on the prescription, design, selection, insertion, maintenance, removal and changing urinary catheters. Health risks associated with urinary catheter misuse are discussed. Urinary catheter use is described with respect to staff training and broader system-level factors. Study participants comprise of LTC home residents 18 years or older and/or LTC staff. Peer-reviewed original research studies of any study design.	Study population comprising of individuals that are not residents of LTC homes. Only children (0-17 years) are included in the study. The sources are study protocols, editorial, conference presentations or letters to the editor. Sources that are not original research such as literature reviews, and/or clinical guidelines/toolkits, systematic reviews and scoping reviews. Studies include animal models/genetic analysis.

**Table A1:** *Search strategy terms and studies retrieved (CINAHL).

**Set**	**Search String**	**Results**
S1	MH “Catheters, Urinary+” OR MH “Urinary Catheterization+” OR MH “Urinary Catheterization, Intermittent"	5780
S2	"Catheters, Urinary” OR “Urinary Catheterization” OR “Urinary Catheterization, Intermittent” OR “Catheter Care, Urinary” OR “Catheter Care, Suprapubic” OR “urinary catheter*” OR “urinary catheterization” OR “urethra* catheter*” OR “intermittent catheter*” OR “foley catheter*” OR “suprapubic catheter*” OR “indwelling catheter*” OR “intermittent catheterization” OR “urethral catheter*” OR “bladder catheter*” OR “catheter insertion” OR “urinary catheterization procedure*” OR “catheter-associated” OR “catheter use” OR “catheter management” OR “transurethral catheter*” OR “suprapubic cystostomy” OR “urinary drainage device*” OR “long-term catheter*” OR “short-term catheter*”	11,651
S3	S1 OR S2	11,702
S4	"nursing home*” OR “care home*” OR “nursing residenc*” OR “nursing care facilit*” OR “nursing care home*” OR “nursing care residenc*” OR “senior citizen* home*” OR “senior citizen* facilit*” OR “senior citizen* residenc*” OR “assisted living facilit*” OR “assisted living home*” OR “assisted living residenc*” OR “assisted living communit*” OR “skilled nursing facilit*” OR “skilled nursing home*” OR “skilled nursing residenc*” OR “longterm care home*” OR “longterm care facilit*” OR “longterm care residen*” OR “long-term care home*” OR “long-term care facilit*” OR “long-term care residen*” OR “convalescent home*” OR “convalescent hospital*” OR “convalescent facilit*” OR “convalescent residenc*” OR “retirement facilit*” OR “retirement home*” OR “retirement residenc*” OR “rest home*” OR “residential care home*” OR “residential care facilit*” OR “home of the aged” OR “homes of the aged” OR “extended care facilit*” OR “extended care home*” OR “old age home*” OR “old age residenc*” OR “old people* home*” OR “old people* residenc*” OR LTCF OR “charitable hom*” OR “charitable facilit*” OR “residential aged care facilities*” OR “aged care homes”	63,255
S5	S3 AND S4	345

* (Search conducted on August 14, 2025)

*Step 3: References List Screening*

The references list of included studies would be screened in addition to the references of relevant systematic reviews, for any additional studies not included through preliminary and full comprehensive search.

#### Concept

In this scoping review, the concept will be the safe and appropriate use of urinary catheters in LTC homes, with emphasis on recommended practices as well as work system characteristics (including people, tasks, technology, physical environment, and organizational factors) that influence catheter-related care. In the context of catheter use in LTC settings, best practices encompass strategies that optimize catheter design, prescription, selection, insertion, removal, and maintenance. These practices help to minimize health complications such as CAUTIs and enhance the quality of life of residents requiring catheterization. For this review, practices will be considered safe, unsafe, recommended, best or evidence-based only when they are described as such in the included literature or relevant guidelines. The reviewers will not independently classify catheter practices into these categories.

#### Context

This scoping review will consider studies from across the globe that are related to the design, prescription, selection, use, management, change and removal of transurethral catheters in LTC homes.

### Types of sources

This scoping review will include (1) original studies employing various study designs including case-controlled, randomized controlled trials, observational, descriptive, experimental, analytical and qualitative studies, (2) studies specifically focusing on the design, prescription, selection, maintenance, removal, change and management of transurethral catheters in LTC home residents 18 years or older, (3) studies published globally in any language will be included during the initial database search. During screening, only articles with full text available in English or French will be included. Letters to the editor, study protocols, and editorials will be excluded. Studies that are not original research such as literature reviews, clinical guidelines/toolkits and narrative reviews will be excluded. A comprehensive search will be conducted across databases from their inception, with no limitations on publication date.

## Methods

This protocol was created by following the Preferred Reporting Items for Systematic Review and Meta-Analysis for Protocols (PRISMA-P) statement (21). Our scoping review will be conducted by following the principles established by Arksey and O’Malley (22). To enhance the rigor and comprehensiveness of our review, we will also employ the guidelines proposed by Peters and colleagues (20, 22) and Levac and colleagues (24). The findings of this scoping review will be reported by using Preferred Reporting Items for Systematic Review and Meta-Analysis extension for Scoping Reviews (PRISMA-ScR) (25). The SEIPS model will be used as an *a priori* analytical framework to guide the extraction and synthesis of findings related to human factors and work-system characteristics influencing catheter use and management in LTC homes.

### Search strategy

This review will adhere to the Joanna Briggs Institute (JBI) guidelines for scoping reviews, incorporating a rigorous three-step search strategy to identify relevant literature (26). In the first step, an initial search of the CINAHL database will be conducted to identify articles on the topic “urinary catheters” and “LTC homes”. This preliminary search will allow for the refinement of the search strategy by examining the key terms in the titles and abstracts of relevant articles, as well as in the index terms used to describe these articles. These insights will inform the development of a comprehensive search string that captures a wide range of relevant studies across databases. In the second step, we will collaborate with the health science librarian (HM) to develop a comprehensive search strategy.

The overall search strategy will include controlled vocabulary, such as MeSH and Emtree, and will also incorporate keywords to account for variations in terminology and indexing across databases.

The finalized search will be conducted across multiple databases, including PubMed, CINAHL (via EBSCO), Embase (via Ovid), MEDLINE (via Ovid), Web of Science and SCOPUS. The third step of the search process will involve hand-searching the reference list of the most relevant eligible articles, such as systematic reviews, to identify additional studies that may not have been retrieved through electronic database search (Appendix I). With this search strategy, we aim to develop a comprehensive understanding of safe urinary catheter use and best practices in LTC homes as stated in the included literature.

### Study selection

Following the comprehensive search, studies will be imported to Covidence systematic review software (Veritas Health Innovation, Melbourne, Australia) wherein the studies will be selected by employing a two-stage selection strategy. The first stage will include title/abstract screening, and the second stage will involve full-text screening, in addition to the removal of duplicates. Each title and abstract will be independently assessed by two reviewers by using the inclusion criteria. At the level of title and abstract screening, studies focusing on the prescription, design, selection, insertion, maintenance, change and, removal of urinary catheters in the LTC setting will be included and eligible studies will move to the full-text screening stage. Each article will be independently reviewed by two authors. Studies that do not specifically focus on urinary catheters in LTC homes, that is if studies specifically focus only on UTIs, CAUTIs or HAIs, will be excluded during full-text review. Conflicts and disagreements arising at each stage of the review process will be discussed and resolved by senior reviewer (AJ) through weekly project check-in meetings. Information on study selection, screening, eligibility and inclusion will be presented in the form of a PRISMA flow diagram generated by Covidence (21).

### Data extraction

Following the full-text screening, essential information will be extracted from the selected studies by developing a comprehensive data chart. Data will be extracted by the first author and senior reviewers (AJ, AC, and CT) will ensure data is extracted and captured coherently. Any conflicts that arise at this stage will be resolved by a senior reviewer (AJ). Data extracted from selected articles will include the name of the author, country and year of publication, age of the study participants, study design, primary aim/purpose and key findings of the study relevant to our research question, with emphasis on information reported on catheter design, prescription, selection, insertion, removal, maintenance and broader system-level factors related to urinary catheter use in the context of LTC homes. We will extract further information from the selected studies in the form of any guidelines/protocols or best practices mentioned for the proper use of urinary catheters in the LTC setting. This could include information about the selection of urinary catheters (based on the materials and ease of use) and strategies to properly maintain and remove them to ensure safe use. In case there is a need for additional information, the authors of the respective articles will be contacted. Missing information will be indicated by “not mentioned”.

### Data analysis and presentation

Following data extraction and charting, the findings will be presented using a descriptive numerical model (20, 22–24). We will not be using any specialized software for data analysis. The extracted data will be summarized through tables and graphs illustrating key variables such as the number of studies by geographic region, publication year, key concepts, and characteristics of the study populations. Additionally, a qualitative thematic analysis will be conducted to identify and synthesize emerging themes. Each included article will be categorized under the relevant themes, allowing for a comprehensive thematic analysis (27–29). Findings related to human factors and work-system characteristics will be organized using the SEIPS model as an *a priori* analytical framework, including the domains of people, tasks, technology, physical environment, and organizational factors. Findings may be mapped to more than one SEIPS domain where interactions between work-system factors are identified. Findings that do not align with the predefined SEIPS domains will be retained and reported as additional themes where appropriate.

This approach will facilitate the extraction of meaningful insights from each study and help to identify gaps in the existing literature. By identifying the gaps in evidence and presenting the advances in this field, we aim to uncover opportunities for standardizing strategies and protocols related to the safe use of urinary catheters in LTC. Finally, we will discuss the potential implications of our findings for future healthcare research and policy development.

## Discussion

Through this systematic scoping review, we aim to map the existing evidence and identify risks, gaps and opportunities in the literature related to the use of urinary catheters in LTC homes. We aim to gather information on the prevalence, outcomes, and clinical indicators associated with urinary catheter use in this setting while identifying evidence-informed practices for catheter prescription, selection, insertion, maintenance, replacement, and removal. By applying the principles of the SEIPS framework, this review will examine how interactions among people, tasks, technology, the physical environment, and organizational factors influence urinary catheter management in LTC settings. By incorporating a human factors and systems perspective, the review will identify work system barriers and facilitators that may affect the implementation of safe catheter practices. This approach has the potential to inform not only clinical decision-making but also improvements in catheter design, clinical workflows, staff education, organizational policies, and implementation strategies. Ultimately, the findings may support the development of evidence-informed policies and interventions tailored to the LTC environment, contributing to improved resident safety and enhanced quality of care.

### Limitations

This scoping review will primarily explore the use and misuse of urinary catheters within LTC settings. Studies conducted in other contexts, such as hospitals, rehabilitation centres or home-care setting, will not be included, which may narrow the scope of the review. As this review will include international literature, differences in terminology used to describe LTC homes, as well as variations in healthcare systems, policies, regulations, and clinical practices, may affect the identification and comparability of relevant studies. These contextual differences will be considered when synthesizing and interpreting the findings. In addition, the rigor and quality of the included studies will not be evaluated.

## Appendix I

Search strategy

Step 1: Initial Limited Search

The initial search was conducted in CINAHL to scope for literature with terms “Urinary Catheter” and “Long-Term care”. This initial limited search was performed to analyze for text words and index terms included in the title and abstract of retrieved article. Insights from the initial limited search were then used to develop a full comprehensive strategy.

*Step 2: Comprehensive Search*

A full comprehensive strategy was formed using insights from the initial search strategy through title and abstract screening with the help of an experienced health sciences librarian.

The finalized search will be conducted across the following databases:

• PubMed

• CINAHL (via EBSCO)

• Embase (via Ovid)

• MEDLINE (via Ovid)

• Web of Science

• SCOPUS

No date or language limits will be applied during the initial searches. However, during screening, only articles with full text available in English or French will be included.

## References

[1] Lachance CC Grobelna A. Management of Patients with Long-Term Indwelling Urinary Catheters: A Review of Guidelines. Ottawa (ON): Canadian Agency for Drugs and Technologies in Health (2019). Available online at: http://www.ncbi.nlm.nih.gov/books/NBK545495/ (CADTH Rapid Response Reports). (Accessed September 14, 2025). 31449368

[2] Gammack JK. Use and management of chronic urinary catheters in long-term care: much controversy, little consensus. J Am Med Direct Assoc. (2002) 3(3):162–8. 10.1016/S1525-8610(04)70459-4. (Accessed September 16, 2025). 12807660

[3] Czwikla J Schmiemann G Hoffmann F. Use of indwelling urinary catheters in nursing home residents: results from a cross-sectional study in 21 German nursing homes. BMC Urol. (2024) 24(1):125. 10.1186/s12894-024-01512-w (Accessed September 16, 2025). 38877475 PMC11177429

[4] Mody L Meddings J Edson BS McNamara SE Trautner BW Stone ND Krein SL Saint S, Enhancing resident safety by preventing healthcare-associated infection: a national initiative to reduce catheter-associated urinary tract infections in nursing homes. Clin Infect Dis. (2015) 61(1):86–94. 10.1093/cid/civ236. (Accessed September 16, 2025). 25814630 PMC4481599

[5] Fiancial Accountability Office of Ontario. Long-Term Care Homes Program: A Review of the Plan to Create 15,000 New Long-Term Care Beds in Ontario - Financial Accountability Office of Ontario %. Financial Accountability Office of Ontario. Available online at: https://fao-on.org/en/report/ontario-long-term-care-program/. (Accessed September 14, 2025)

[6] Statista. Number of long-term care homes by province Canada 2021. Available online at: https://www.statista.com/statistics/1441145/number-ltc-home-canada-by-province/. (Accessed September 14, 2025).

[7] Nicolle LE SHEA Long-Term-Care-Committee. Urinary tract infections in long-term-care facilities. Infect Control Hosp Epidemiol 2001;22:167–75. 10.1086/501886 11310697

[8] Schaeffer AJ Richie JP Chen W. Placement and management of urinary bladder catheters in adults. (2017). Available online at: https://doctorabad.com/app/uptodate/d/topic.htm?path=placement-and-management-of-urinary-bladder-catheters-in-adults. (Accessed September 16, 2025)

[9] Gray M Skinner C Kaler W. External collection devices as an alternative to the indwelling urinary catheter. J Wound Ostomy Continence Nurs. (2016) 43(3):301–7. 10.1097/WON.0000000000000220. (Accessed September 16, 2025). 26974963 PMC4870965

[10] Prieto JA Murphy CL Stewart F Fader M. Intermittent catheter techniques, strategies and designs for managing long-term bladder conditions. Cochrane Database Syst Rev. (2021) 2021(10):CD006008. Available online at: https://www.ncbi.nlm.nih.gov/pmc/articles/PMC8547544/ (Accessed September 16, 2025). 10.1002/14651858.CD006008.pub5 PMC8547544 34699062

[11] Corder CJ LaGrange CA. Suprapubic bladder catheterization. In: StatPearls. Treasure Island (FL): StatPearls Publishing. (2025). Available online at: http://www.ncbi.nlm.nih.gov/books/NBK482179/ (Accessed September 14, 2025).

[12] Czwikla J Wandscher K Helbach J Fassmer AM Schmiemann G Hoffmann F. Prevalence of indwelling urinary catheters in nursing home residents: systematic review. Int J Nurs Stud. (2023) 145:104555. 10.1016/j.ijnurstu.2023.104555. (Accessed September 16, 2025). 37421830

[13] Genao L Buhr GT. Urinary tract infections in older adults residing in long-term care facilities. Ann Longterm Care. (2012) 20(4):33–8. Available online at: https://www.ncbi.nlm.nih.gov/pmc/articles/PMC3573848/. (Accessed September 16, 2025). 23418402 PMC3573848

[14] Bhatt NR Davis NF Thorman H Brierly R Scopes J. Knowledge, skills, and confidence among healthcare staff in urinary catheterization. Can Urol Assoc J. (2021) 15(9):E488–94. 10.5489/cuaj.6986 33591901 PMC8490097

[15] CDC. Urinary Tract Infection. (2025). Clinical Safety: Preventing Catheter-associated Urinary Tract Infections (CAUTIs). Available online at: https://www.cdc.gov/uti/hcp/clinical-safety/index.html (Accessed Sept 14, 2025).

[16] Prieto J Wilson J Bak A Denton A Flores A Lusardi G. A prevalence survey of patients with indwelling urinary catheters on district nursing caseloads in the United Kingdom: the community urinary catheter management (CCaMa) study. J Infect Prev. (2020) 21(4):129–35. 10.1177/1757177420901550 32655693 PMC7328505

[17] Long-term care homes in Canada: How many and who owns them? | CIHI [Internet]. Available online at: https://www.cihi.ca/en/long-term-care-homes-in-canada-how-many-and-who-owns-them (Accessed 2025 Sept 14).

[18] McGrail KM McGregor MJ Cohen M Tate RB Ronald LA. For-profit versus not-for-profit delivery of long-term care. CMAJ. (2007) 176(1):57–8. 10.1503/cmaj.060591 17200392 PMC1764560

[19] Harvard Health [Internet]. (2019). Urinary Catheterization. Available online at: https://www.health.harvard.edu/staying-healthy/urinary-catheterization-a-to-z (Accessed Sept 14, 2025).

[20] Peters MDJ Godfrey CM Khalil H McInerney P Parker D Soares CB. Guidance for conducting systematic scoping reviews. Int J Evid Based Healthc. (2015) 13(3):141–6. 10.1097/XEB.0000000000000050 26134548

[21] Moher D Shamseer L Clarke M Ghersi D Liberati A Petticrew M. Preferred reporting items for systematic review and meta-analysis protocols (PRISMA-P) 2015 statement. Syst Rev. (2015) 4(1):1. 10.1186/2046-4053-4-1 25554246 PMC4320440

[22] Arksey H O’Malley L. Scoping studies: towards a methodological framework. Int J Soc Res Methodol. (2005) 8(1):19–32. 10.1080/1364557032000119616

[23] Peters MDJ Marnie C Tricco AC Pollock D Munn Z Alexander L. Updated methodological guidance for the conduct of scoping reviews. JBI Evid Synth. (2020) 18(10):2119–26. 10.11124/JBIES-20-00167 33038124

[24] Levac D Colquhoun H O’Brien KK. Scoping studies: advancing the methodology. Implement Sci. (2010) 5(1):69. 10.1186/1748-5908-5-69 20854677 PMC2954944

[25] Tricco AC Lillie E Zarin W O'Brien KK Colquhoun H Levac D. PRISMA extension for scoping reviews (PRISMA-ScR): checklist and explanation. Ann Intern Med. (2018) 169(7):467–73. 10.7326/M18-0850 30178033

[26] Dos Santos WM Secoli SR Püschel VAA. The joanna briggs institute approach for systematic reviews. Rev Lat Am Enfermagem. (2018) 26:e3074. 10.1590/1518-8345.2885.3074 30462787 PMC6248737

[27] Vaismoradi M Jones J Turunen H Snelgrove S. Theme development in qualitative content analysis and thematic analysis. JNEP. (2016) 6(5):100. 10.5430/jnep.v6n5p100

[28] (PDF) Using thematic analysis in psychology. ResearchGate [Internet]. (2025) Available online at: https://www.researchgate.net/publication/235356393_Using_thematic_analysis_in_psychology (Accessed 2025 Sept 16).

[29] Guest G MacQueen KM Namey EE. Applied Thematic Analysis. SAGE Publications, Inc. (2012). Available online at: https://methods.sagepub.com/book/mono/applied-thematic-analysis/toc (Accessed 2025 Sept 14).

[30] (PDF) Urinary Catheter Change Frequency Infection Prevention and Control Communication Form. [Internet]; Available online at: https://professionals.wrha.mb.ca/old/extranet/ipc/files/manuals/community/7.2.3.pdf (Accessed April 2, 2026).

[31] Infection control guidelines for long-term care facilities. Health Canada [Internet]. (1995) Available online at: https://publications.gc.ca/collections/collection_2025/sc-hc/H30-11-6-6-1994-eng.pdf. (Accessed April 2, 2026).

[32] Carayon P Schoofs Hundt A Karsh B-T Gurses AP Alvarado CJ Smith M. Work system design for patient safety: the SEIPS model. Qual Saf Health Care. (2006) 15:i50–8. 10.1136/qshc.2005.015842 17142610 PMC2464868

[33] Yadav M Kumar A Bharti S. Polymicrobial biofilms and catheter-associated UTIs: emerging clinical challenges and management perspectives. Next Nanotechnology. (2026) 10:100679. 10.1016/j.nxnano.2026.100679

